# Crystal structure of a methimazole-based ionic liquid

**DOI:** 10.1107/S2056989015022136

**Published:** 2015-12-06

**Authors:** Jamie C. Gaitor, Manuel Sanchez Zayas, Darrel J. Myrthil, Frankie White, Jeffrey M. Hendrich, Richard E. Sykora, Richard A. O’Brien, John T. Reilly, Arsalan Mirjafari

**Affiliations:** a Department of Chemistry and Physics, Florida Gulf Coast University, Fort Myers, FL 33965, USA; b University of South Alabama, Department of Chemistry, Mobile, AL 36688, USA

**Keywords:** crystal structure, ionic liquids, methimazole, *S*-allyl­ation, nitro­gen heterocycle

## Abstract

The structure of 1-methyl-2-(prop-2-en-1-ylsulfan­yl)-1*H*-imidazol-3-ium bromide, C_7_H_11_N_2_S^+^·Br^−^, has monoclinic (*P*2_1_/*c*) symmetry. In the crystal, the components are linked by N—H⋯Br and C—H⋯Br hydrogen bonds. The crystal structure of the title compound undeniably proves that methimazole reacts through the thione tautomer, rather than the thiol tautomer in this system.

## Related literature   

For the biological activity of methimazole, see: Rong *et al.* (2013 ▸). For its use as a ligand, see: Crossley *et al.* (2006 ▸). For a discussion of methimazole-based ionic liquids, see: Siriwardana *et al.* (2008 ▸). For reaction chemistry of methimazole, see: Roy & Mugesh (2005 ▸).
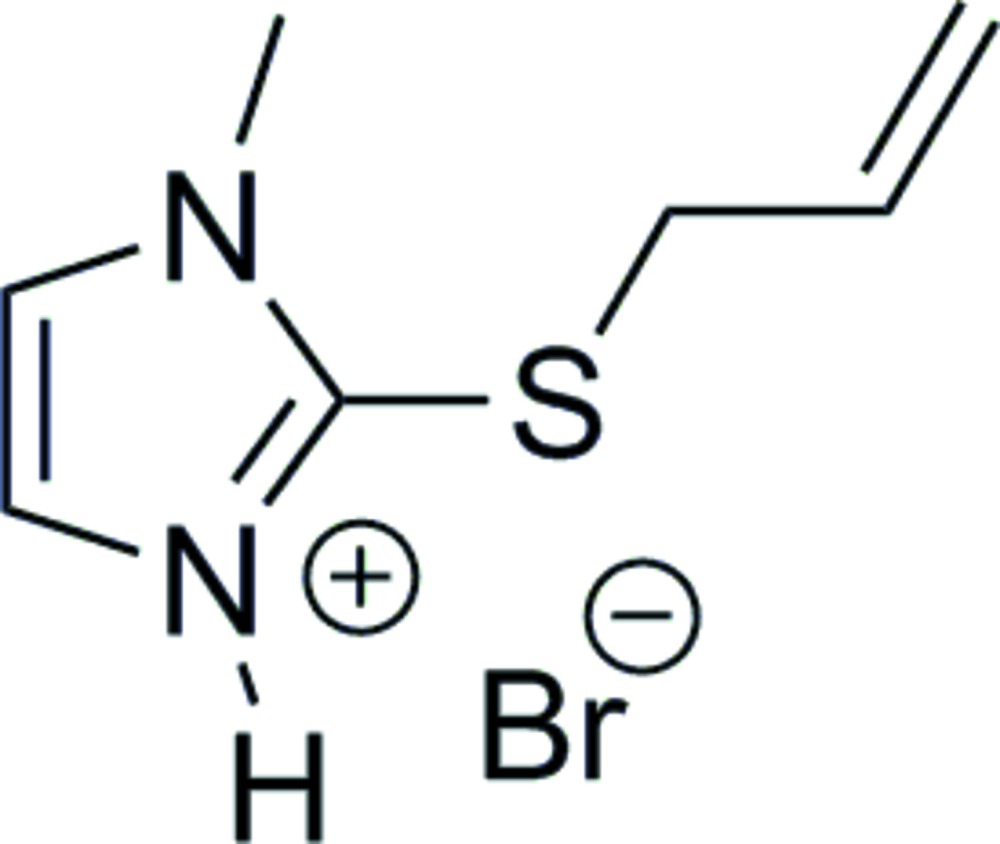


## Experimental   

### Crystal data  

C_7_H_11_N_2_S^+^·Br^−^
*M* *_r_* = 235.15
Monoclinic, 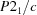

*a* = 10.8692 (7) Å
*b* = 7.4103 (5) Å
*c* = 12.8551 (9) Å
β = 104.006 (7)°
*V* = 1004.62 (11) Å^3^
*Z* = 4
Mo *K*α radiation
μ = 4.24 mm^−1^
*T* = 180 K
0.6 × 0.32 × 0.25 mm

### Data collection  

Agilent Xcalibur, Eos diffractometer
Absorption correction: multi-scan (*CrysAlis PRO*; Agilent, 2014 ▸) *T*_min_ = 0.321, *T*_max_ = 1.000
7388 measured reflections
1829 independent reflections
1558 reflections with *I* > 2σ(*I*)
*R*_int_ = 0.042

### Refinement  

*R*[*F* ^2^ > 2σ(*F* ^2^)] = 0.030
*wR*(*F* ^2^) = 0.065
*S* = 1.03
1829 reflections
105 parameters
1 restraint
H atoms treated by a mixture of independent and constrained refinement
Δρ_max_ = 0.34 e Å^−3^
Δρ_min_ = −0.38 e Å^−3^

### 

Data collection: *CrysAlis PRO* (Agilent, 2014 ▸); cell refinement: *CrysAlis PRO*; data reduction: *CrysAlis PRO*; program(s) used to solve structure: *SHELXS97* (Sheldrick, 2008 ▸); program(s) used to refine structure: *SHELXL97* (Sheldrick, 2008 ▸); molecular graphics: *OLEX2* (Dolomanov *et al.*, 2009 ▸); software used to prepare material for publication: *OLEX2* and *publCIF* (Westrip, 2010 ▸).

## Supplementary Material

Crystal structure: contains datablock(s) I, New_Global_Publ_Block. DOI: 10.1107/S2056989015022136/hg5463sup1.cif

Structure factors: contains datablock(s) I. DOI: 10.1107/S2056989015022136/hg5463Isup2.hkl

Supporting information file. DOI: 10.1107/S2056989015022136/hg5463Isup3.cml

**Fig. 1.**
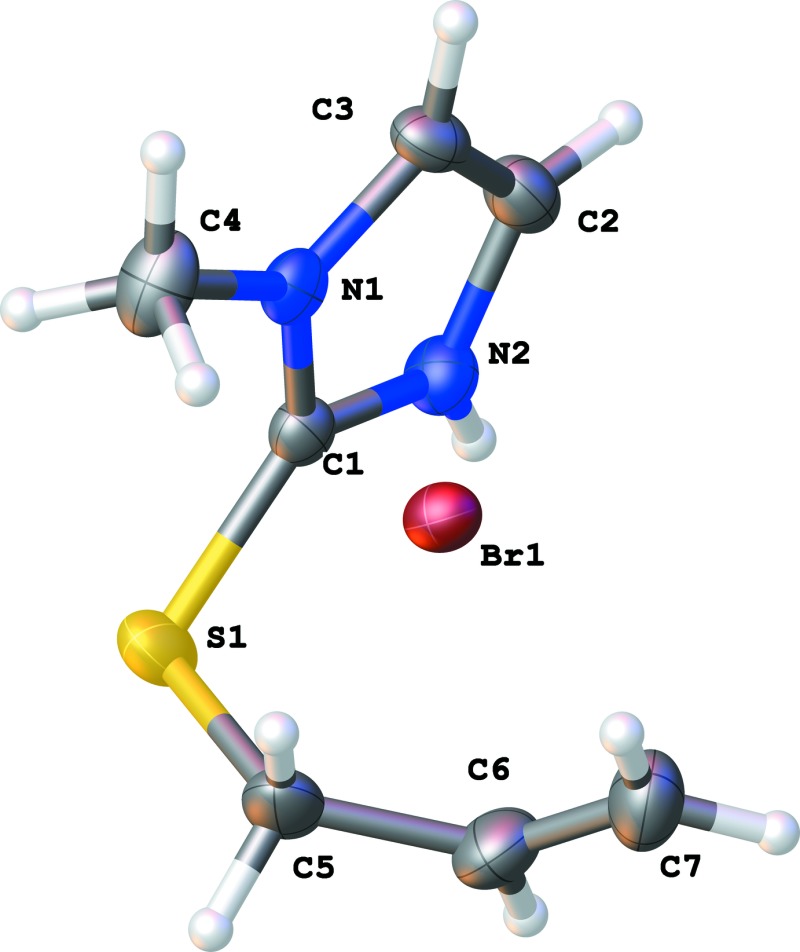
A thermal ellipsoid diagram of the structure of the title compound.

. DOI: 10.1107/S2056989015022136/hg5463fig1.tif
A thermal ellipsoid diagram of the structure of the title compound.

**Fig. 2.**
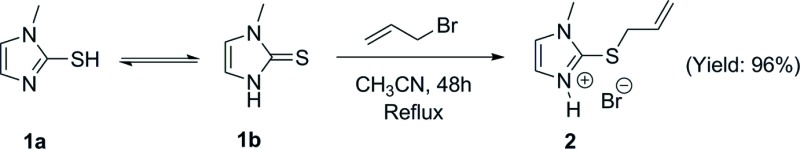
Reaction scheme.

. DOI: 10.1107/S2056989015022136/hg5463fig2.tif
Reaction scheme.

CCDC reference: 1437865

Additional supporting information:  crystallographic information; 3D view; checkCIF report

## Figures and Tables

**Table 1:** Hydrogen-bond geometry (Å, °)

*D*—H⋯*A*	*D*—H	H⋯*A*	*D*⋯*A*	*D*—H⋯*A*
N2—H2⋯Br1^i^	0.84 (3)	2.46 (3)	3.246 (2)	158 (3)
C2—H2*A*⋯Br1^ii^	0.93	2.84	3.723 (4)	159
C3—H3⋯Br1^iii^	0.93	2.91	3.757 (3)	152
C4—H4*B*⋯Br1	0.96	2.87	3.737 (3)	151
C5—H5*B*⋯Br1^iv^	0.97	2.89	3.814 (3)	161

Symmetry codes: (i) 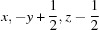; (ii) 
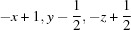
; (iii) 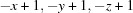; (iv) 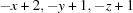.

## supplementary crystallographic information

### S1. Comment

2-Mercapto-1-methyl­imidazole or methimazole 1 belongs to a class of five-membered heterocyclic nitro­gen compounds, which possess various biological activities (e.g. it is a widely used anti-thyroid drug under the name Tapazole), see: Rong *et al.* (2013). Additionally, it has found use as a multidentate ligand in the fields of inorganic and organometallic chemistry, in which the sulfur atom can serve as a soft donor towards a wide variety of transition metals, see: Crossley *et al.* (2006). The alkyl­ation of methimazole with alkyl halides (e.g. iodo­ethane and chloro­butane) lead to the formation of methimazole-based ionic liquids in high yields, see Siriwardana *et al.* (2008). To date, no methimazole-based ionic liquids have been structurally characterized by X-ray diffraction.

Methimazole exists in two tautomeric forms, equilibrating between the 2-thiol 1a and 2-thione 1b, and both N-alkyl­ation and S-alkyl­ation reactions are possible, depending upon the reaction conditions and types of substrates employed, see Roy & Mugesh (2005). They reported that only S-alkyl­ated methimazoles were formed. The product structures were established by NMR spectroscopy, which is elusive in terms of proving the exclusive formation of S-alkyl­ated products over N-alkyl­ated products. Herein, we report the crystal structure of S-allyl­ated methimazolium bromide 2, which was prepared in qu­anti­tative yield (96%) via the reaction of methimazole with allyl bromide in refluxing aceto­nitrile (Scheme S1). The crystal structure of 2 undeniably proves that methimazole reacts through the 2-thione tautomer 1b.

### S2. Synthesis and crystallization

2-Mercapto-1-methyl­imidazole (0.57 g, 5 mmol) and allyl bromide (0.85 g, 7 mmol) were dissolved in aceto­nitrile (5.0 mL) and the mixture refluxed for 48 hours. The solvent and excess allyl bromide were removed under vacuum to afford an off-white solid. The solid was washed with toluene (3 x 10 mL) and then recrystallized in aceto­nitrile to yield pure product 2 as an off-white solid in 96% isolated yield.

### S3. Refinement

Crystal data, data collection and structure refinement details are summarized in Table 1. The H-atom (H2) located on N2 was allowed to freely refine (isotropically). The remaining H-atoms were placed in calculated positions and allowed to ride during subsequent refinement, with *U*_iso_(H) = 1.5*U*_eq_(C) and C—H distances of 0.96 Å for methyl hydrogens, with *U*_iso_(H) = 1.2*U*_eq_(C) and C—H distances of 0.97 Å for the secondary hydrogens, and with *U*_iso_(H) = 1.2*U*_eq_(C) and C—H distances of 0.93 Å for all remaining hydrogen atoms.

### Crystal data

**Table:**

C_7_H_11_N_2_S^+^·Br^−^	*F*(000) = 472
*M**_r_* = 235.15	*D*_x_ = 1.555 Mg m^−^^3^
Monoclinic, *P*2_1_/*c*	Mo *K*α radiation, λ = 0.71073 Å
*a* = 10.8692 (7) Å	Cell parameters from 2203 reflections
*b* = 7.4103 (5) Å	θ = 3.9–27.0°
*c* = 12.8551 (9) Å	µ = 4.24 mm^−^^1^
β = 104.006 (7)°	*T* = 180 K
*V* = 1004.62 (11) Å^3^	Prism, colourless
*Z* = 4	0.6 × 0.32 × 0.25 mm

### Data collection

**Table:**

Agilent Xcalibur, Eos diffractometer	1829 independent reflections
Radiation source: Enhance (Mo) X-ray Source	1558 reflections with *I* > 2σ(*I*)
Graphite monochromator	*R*_int_ = 0.042
Detector resolution: 16.0514 pixels mm^-1^	θ_max_ = 25.3°, θ_min_ = 3.2°
ω scans	*h* = −13→13
Absorption correction: multi-scan (*CrysAlis PRO*; Agilent, 2014)	*k* = −8→8
*T*_min_ = 0.321, *T*_max_ = 1.000	*l* = −15→15
7388 measured reflections	

### Refinement

**Table:**

Refinement on *F*^2^	Primary atom site location: structure-invariant direct methods
Least-squares matrix: full	Secondary atom site location: difference Fourier map
*R*[*F*^2^ > 2σ(*F*^2^)] = 0.030	Hydrogen site location: inferred from neighbouring sites
*wR*(*F*^2^) = 0.065	H atoms treated by a mixture of independent and constrained refinement
*S* = 1.03	*w* = 1/[σ^2^(*F*_o_^2^) + (0.027*P*)^2^] where *P* = (*F*_o_^2^ + 2*F*_c_^2^)/3
1829 reflections	(Δ/σ)_max_ = 0.001
105 parameters	Δρ_max_ = 0.34 e Å^−^^3^
1 restraint	Δρ_min_ = −0.38 e Å^−^^3^

### Special details

**Table:**

Experimental. *CrysAlis Pro* (Agilent, 2014) Empirical absorption correction using spherical harmonics, implemented in SCALE3 ABSPACK scaling algorithm.
Geometry. All e.s.d.'s (except the e.s.d. in the dihedral angle between two l.s. planes) are estimated using the full covariance matrix. The cell e.s.d.'s are taken into account individually in the estimation of e.s.d.'s in distances, angles and torsion angles; correlations between e.s.d.'s in cell parameters are only used when they are defined by crystal symmetry. An approximate (isotropic) treatment of cell e.s.d.'s is used for estimating e.s.d.'s involving l.s. planes.
Refinement. Refinement of *F*^2^ against ALL reflections. The weighted *R*-factor *wR* and goodness of fit *S* are based on *F*^2^, conventional *R*-factors *R* are based on *F*, with *F* set to zero for negative *F*^2^. The threshold expression of *F*^2^ > 2σ(*F*^2^) is used only for calculating *R*-factors(gt) *etc*. and is not relevant to the choice of reflections for refinement. *R*-factors based on *F*^2^ are statistically about twice as large as those based on *F*, and *R*- factors based on ALL data will be even larger.

### Fractional atomic coordinates and isotropic or equivalent isotropic displacement parameters (Å^2^)

**Table:**

	*x*	*y*	*z*	*U*_iso_*/*U*_eq_	
Br1	0.70880 (3)	0.66161 (4)	0.53045 (2)	0.02621 (12)	
N2	0.6562 (2)	0.0414 (3)	0.2392 (2)	0.0273 (6)	
N1	0.6302 (2)	0.1226 (3)	0.39296 (18)	0.0233 (6)	
C2	0.5297 (3)	0.0739 (4)	0.2258 (3)	0.0316 (8)	
H2A	0.4669	0.0623	0.1625	0.038*	
C3	0.5134 (3)	0.1262 (4)	0.3221 (3)	0.0285 (7)	
H3	0.4371	0.1587	0.3376	0.034*	
C1	0.7171 (3)	0.0704 (4)	0.3410 (2)	0.0234 (7)	
S1	0.87834 (8)	0.04183 (11)	0.39532 (7)	0.0373 (2)	
C4	0.6547 (4)	0.1637 (4)	0.5076 (2)	0.0370 (9)	
H4A	0.5756	0.1749	0.5278	0.055*	
H4B	0.7009	0.2750	0.5221	0.055*	
H4C	0.7037	0.0681	0.5481	0.055*	
C6	0.9092 (3)	0.3747 (4)	0.3027 (3)	0.0410 (9)	
H6	0.9450	0.3274	0.2498	0.049*	
C5	0.9322 (3)	0.2790 (4)	0.4066 (3)	0.0405 (9)	
H5A	0.8889	0.3429	0.4532	0.049*	
H5B	1.0223	0.2818	0.4401	0.049*	
C7	0.8417 (3)	0.5215 (5)	0.2809 (3)	0.0436 (9)	
H7A	0.8047	0.5719	0.3323	0.052*	
H7B	0.8305	0.5758	0.2141	0.052*	
H2	0.689 (3)	0.007 (4)	0.190 (2)	0.049 (11)*	

### Atomic displacement parameters (Å^2^)

**Table:**

	*U*^11^	*U*^22^	*U*^33^	*U*^12^	*U*^13^	*U*^23^
Br1	0.02573 (18)	0.0284 (2)	0.02529 (19)	−0.00350 (13)	0.00772 (13)	−0.00185 (13)
N2	0.0309 (15)	0.0295 (16)	0.0227 (15)	0.0016 (12)	0.0088 (13)	−0.0005 (12)
N1	0.0304 (15)	0.0165 (13)	0.0238 (14)	−0.0003 (11)	0.0081 (12)	0.0002 (10)
C2	0.0256 (17)	0.030 (2)	0.0354 (19)	−0.0008 (14)	−0.0003 (15)	0.0026 (15)
C3	0.0190 (16)	0.0255 (18)	0.041 (2)	0.0028 (13)	0.0064 (14)	0.0023 (15)
C1	0.0253 (17)	0.0180 (17)	0.0264 (17)	−0.0015 (13)	0.0057 (14)	0.0027 (13)
S1	0.0229 (4)	0.0335 (5)	0.0518 (6)	0.0031 (4)	0.0019 (4)	0.0112 (4)
C4	0.059 (2)	0.028 (2)	0.0254 (18)	−0.0016 (16)	0.0128 (17)	−0.0035 (14)
C6	0.039 (2)	0.045 (2)	0.044 (2)	−0.0026 (17)	0.0186 (18)	0.0108 (18)
C5	0.0249 (18)	0.040 (2)	0.051 (2)	−0.0105 (15)	−0.0023 (16)	0.0134 (17)
C7	0.049 (2)	0.042 (2)	0.037 (2)	0.0000 (18)	0.0056 (18)	0.0126 (17)

### Geometric parameters (Å, º)

**Table:**

N2—C2	1.366 (4)	C4—H4A	0.9600
N2—C1	1.334 (4)	C4—H4B	0.9600
N2—H2	0.836 (17)	C4—H4C	0.9600
N1—C3	1.373 (4)	C6—H6	0.9300
N1—C1	1.338 (3)	C6—C5	1.480 (4)
N1—C4	1.465 (4)	C6—C7	1.304 (4)
C2—H2A	0.9300	C5—H5A	0.9700
C2—C3	1.349 (4)	C5—H5B	0.9700
C3—H3	0.9300	C7—H7A	0.9300
C1—S1	1.736 (3)	C7—H7B	0.9300
S1—C5	1.847 (3)		
			
C2—N2—H2	124 (2)	N1—C4—H4B	109.5
C1—N2—C2	109.9 (3)	N1—C4—H4C	109.5
C1—N2—H2	126 (2)	H4A—C4—H4B	109.5
C3—N1—C4	125.3 (3)	H4A—C4—H4C	109.5
C1—N1—C3	109.0 (2)	H4B—C4—H4C	109.5
C1—N1—C4	125.7 (3)	C5—C6—H6	118.1
N2—C2—H2A	126.7	C7—C6—H6	118.1
C3—C2—N2	106.7 (3)	C7—C6—C5	123.8 (3)
C3—C2—H2A	126.7	S1—C5—H5A	108.8
N1—C3—H3	126.3	S1—C5—H5B	108.8
C2—C3—N1	107.3 (3)	C6—C5—S1	113.8 (2)
C2—C3—H3	126.3	C6—C5—H5A	108.8
N2—C1—N1	107.1 (3)	C6—C5—H5B	108.8
N2—C1—S1	126.0 (2)	H5A—C5—H5B	107.7
N1—C1—S1	126.9 (2)	C6—C7—H7A	120.0
C1—S1—C5	100.68 (14)	C6—C7—H7B	120.0
N1—C4—H4A	109.5	H7A—C7—H7B	120.0
			
N2—C2—C3—N1	−0.7 (3)	C1—N2—C2—C3	0.7 (4)
N2—C1—S1—C5	104.2 (3)	C1—N1—C3—C2	0.5 (3)
N1—C1—S1—C5	−77.0 (3)	C1—S1—C5—C6	−61.4 (3)
C2—N2—C1—N1	−0.4 (3)	C4—N1—C3—C2	−177.7 (3)
C2—N2—C1—S1	178.7 (2)	C4—N1—C1—N2	178.1 (3)
C3—N1—C1—N2	−0.1 (3)	C4—N1—C1—S1	−0.9 (4)
C3—N1—C1—S1	−179.1 (2)	C7—C6—C5—S1	121.8 (3)

### Hydrogen-bond geometry (Å, º)

**Table:**

*D*—H···*A*	*D*—H	H···*A*	*D*···*A*	*D*—H···*A*
N2—H2···Br1^i^	0.84 (3)	2.46 (3)	3.246 (2)	158 (3)
C2—H2*A*···Br1^ii^	0.93	2.84	3.723 (4)	159
C3—H3···Br1^iii^	0.93	2.91	3.757 (3)	152
C4—H4*B*···Br1	0.96	2.87	3.737 (3)	151
C5—H5*B*···Br1^iv^	0.97	2.89	3.814 (3)	161

Symmetry codes: (i) *x*, −*y*+1/2, *z*−1/2; (ii) −*x*+1, *y*−1/2, −*z*+1/2; (iii) −*x*+1, −*y*+1, −*z*+1; (iv) −*x*+2, −*y*+1, −*z*+1.
