# Supplementary figures and images for: Crystal structure of a methimazole-based ionic liquid
Source: Acta Crystallogr E Crystallogr Commun. 2015 Dec 6;71(Pt 12):o1008–9. doi: 10.1107/S2056989015022136 (PMC4719949; doi:10.1107/S2056989015022136)

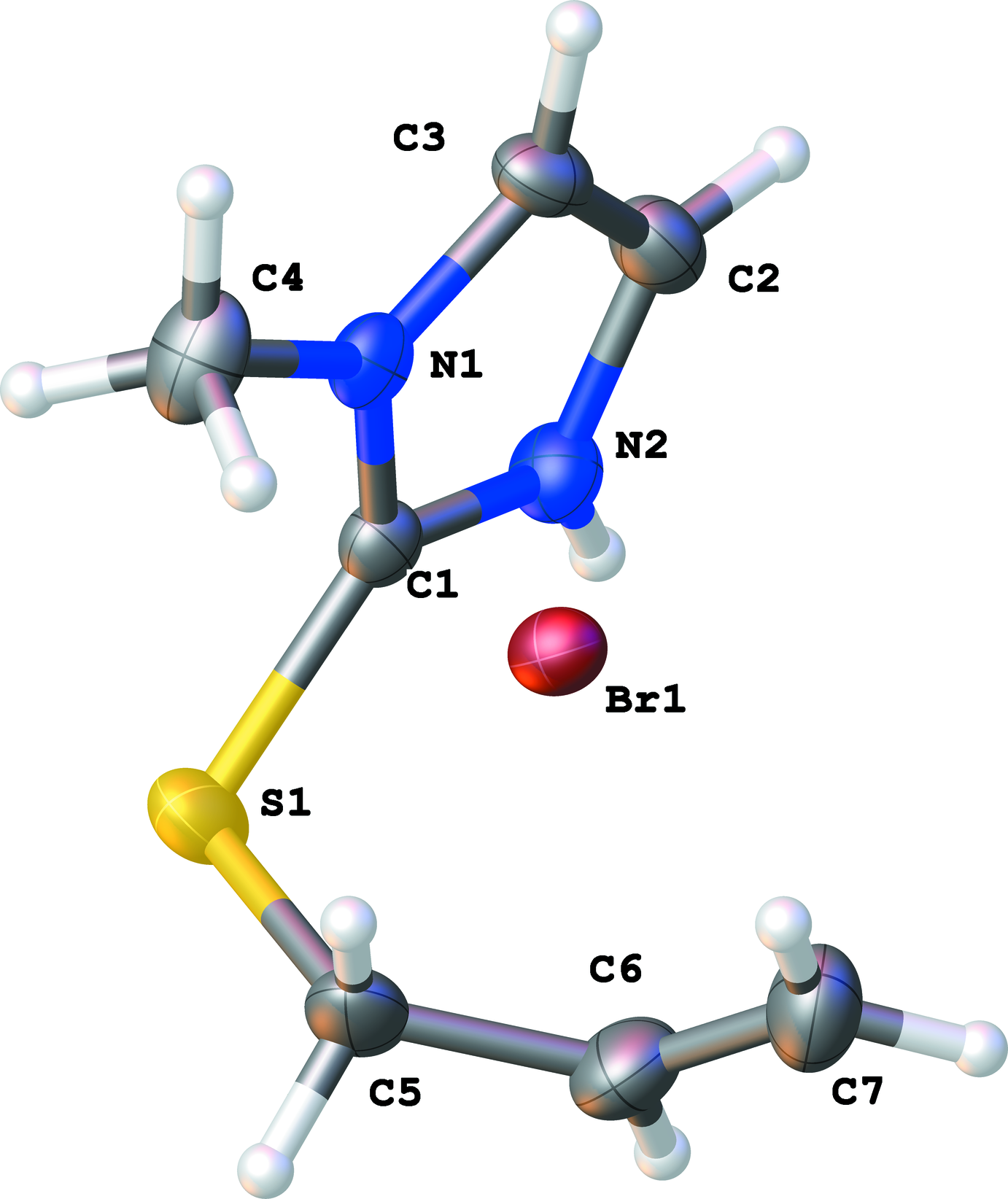

Supplement: Supplementary file 4 [file e-71-o1008-fig1.tif]

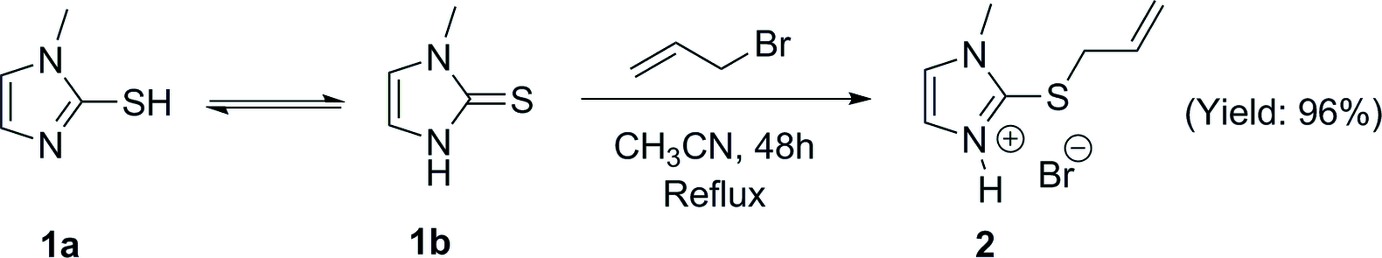

Supplement: Supplementary file 5 [file e-71-o1008-fig2.tif]
